# Diabetic Hearts Exhibit Global DNA Hypermethylation That Alter the Mitochondrial Functional Genes to Enhance the Sensitivity of the Heart to Ischemia Reperfusion Injury

**DOI:** 10.3390/biomedicines10123065

**Published:** 2022-11-28

**Authors:** Sri Rahavi Boovarahan, David Raj Chellappan, Nemat Ali, Abdullah F. AlAsmari, Mohammad Waseem, Abdullah Saad Alabdulrahim, Ziyad Ali Alzahrani, Gino A. Kurian

**Affiliations:** 1 Vascular Biology Laboratory, School of Chemical and Biotechnology, SASTRA Deemed University, Thanjavur 613401, India; 2 School of Chemical and Biotechnology, SASTRA Deemed University, Thanjavur 613401, India; 3 Department of Pharmacology and Toxicology, College of Pharmacy, King Saud University, P.O. Box 55760, Riyadh 11451, Saudi Arabia; 4 Department of Pharmaceutical Sciences, University of Maryland Eastern Shore School of Pharmacy, Princess Anne, MD 21853, USA

**Keywords:** diabetes, ischemia reperfusion, mitochondria, mRNA expression, DNA methylation

## Abstract

A recent study has shown that DNA hypermethylation can promote ischemia reperfusion (I/R) injury by regulating the mitochondrial function. Diabetes mellitus (DM) is reported to induce DNA hypermethylation, but whether this prior DNA methylation in DM I/R heart inflicts a beneficial or detrimental effect is not known and is addressed in this study. DM was induced in 6-week-old male Wistar rats with streptozotocin (65 mg/kg b.wt). After 24 weeks on a normal diet, I/R was induced in rat heart using a Langendorff perfusion system and analyzed the myocardium for different parameters to measure hemodynamics, infarct size, DNA methylation and mitochondrial function. Diabetic heart exhibited DNA hypermethylation of 39% compared to the control, along with DNMT expression elevated by 41%. I/R induction in diabetic heart promoted further DNA hypermethylation (24%) with aggravated infarct size (21%) and reduced the cardiac rate pressure product (43%) from I/R heart. Importantly, diabetic I/R hearts also experienced a decline in the mitochondrial copy number (60%); downregulation in the expression of mitochondrial bioenergetics (*ND1*, *ND2*, *ND3*, *ND4*, *ND5*, *ND6*) and mitofusion (*MFN1*, *MFN2*) genes and the upregulation of mitophagy (*PINK*, *PARKIN*, *OPTN*) and mitofission (*MFF*, *DNM1*, *FIS1*) genes that reduce the dp/dt contribute to the contractile dysfunction in DM I/R hearts. Besides, a negative correlation was obtained between mitochondrial *PGC1α*, *POLGA*, *TFAM* genes and DNA hypermethylation in DM I/R hearts. Based on the above data, the elevated global DNA methylation level in diabetic I/R rat hearts deteriorated the mitochondrial function by downregulating the expression of *POLGA*, *TFAM* and *PGC1α* genes and negatively contributed to I/R-associated increased infarct size and altered hemodynamics.

## 1. Introduction

DNA methylation can alter the gene expression without changing the DNA sequence, where a methyl group is added to cytosine residues present primarily in CpG islands of the promoter regions of genes. This process is catalyzed by a set of DNA methyltransferase (DNMT) enzymes and regulated by a battery of ten eleven translocase (TET) genes. Recent studies have utilized DNA methylation as a potential biomarker for the identification of metabolic diseases like type II diabetes mellitus (DM) [1] and cardiovascular disease [2].

Global DNA methylation and gene-specific methylation provide mixed results in DM, wherein few studies reported hypomethylation, while a few others suggested the hypermethylation of DNA. For instance, a recent study by Marumo and his group (2015) reported the aberrant hypomethylation of *Agt*, *Cyp4a10*, *Glut5* and *Met* in proximal tubular cells in diabetic kidney [3], while an another study conducted in Han Chinese individuals have shown that the hypermethylation of nuclear receptor subfamily 4 group can increase the susceptibility to type II DM [4] This difference may be due to variation in the study population or different conditions of DM and techniques used in the study. This discrepancy can be addressed to a greater extent in preclinical study, wherein experimental conditions are homogenous. Moreover, methylation levels are tissue-specific. For instance, age-associated hypomethylation was exhibited in heart, liver and intestine, while the kidney experienced age-associated hypermethylation. Further, recent genome-wide gene-specific DNA methylation data reveal that the impact of diabetes-linked methylation is not similar in every tissue. 

Diabetic patients are susceptible to ischemic insult due to altered metabolism, thus promoting the development of ischemic heart disease. The standard protocol for the management of ischemic patients include a revascularization procedure that often leads to another injury named reperfusion injury [5]. Limited evidence is available in the literature that explains the consequence of basal epigenetic alterations in DM heart in the recovery efficiency of heart from the revascularization protocol. 

Ischemic reperfusion (I/R) conditions can alter the DNA methylation by influencing the expression of *DNMTs* and the *TETs* (eraser of methylation) gene, which was evident from the I/R study in kidney, brain and heart [6,7,8]. In focal cerebral ischemia reperfusion injury model, investigators have shown that focal ischemia increased 5 hmc at the promoter region of thousands of genes that adversely affect the cell survival, angiogenesis, neurogenesis, antioxidant defence and DNA repair. A recent study explained the promotion of hypermethylation during myocardial I/R by activating DNMT and suppressing TET enzymes [8]. Further, the same group provided evidence for the association of global DNA methylation and mitochondrial function in I/R rat heart. However, DM rat heart is already reported to possess an altered global DNA methylation profile and to possess low mitochondrial functional activities [9]. However, not much information is available in the literature that explains the impact of I/R on diabetic hearts that exhibit basal tissue level modification. The I/R-associated alterations in the DNA methylation profile and their subsequent influence on the mitochondrial function (Key player of I/R pathology) are not known. 

In the present study, we evaluated the global DNA methylation level and subsequent enzymes involved in diabetic rat heart and its aftermath of ischemia reperfusion injury. Further, we evaluated its impact on the mediator of I/R, especially, the mitochondrial function from gene perception.

## 2. Materials and Methods

### 2.1. Animals

All procedures involving the animals were reviewed and approved by the Institutional Animal Ethics Committee (IAEC), SASTRA University, Thanjavur, India (CPCSEA Approval No. 300/SASTRA/IAEC/RPP) and was conducted in accordance with the CPCSEA (Committee for the Purpose of Control and Supervision of Experiments on Animals) guidelines. Animals were housed in polycarbonate cages and maintained at 25 ± 2 °C with a 12 h light/dark cycle and relative humidity of 65 ± 2%. Feed and water were provided ad libitum.

### 2.2. Experimental Groups

Male Wistar rats (200–250 g) 24 weeks old were divided into 4 experimental groups on a random basis (n = 6/group): (1) normal (N), (2) ischemia reperfusion (I/R), (3) diabetes control (DM-C), (4) diabetes+ischemia reperfusion (DM-I/R). 

The rats were anaesthetized with sodium thiopentone (60 mg/kg b.wt. i.p.). The hearts were excised and mounted on a Langendorff apparatus (AD Instruments, Sydney, Australia) and perfused with Krebs–Henseleit (KH) buffer in a constant pressure mode as per the treatment groups. 

The rats in the N and DM-C groups were perfused continuously with KH buffer for 120 min. For the I/R and DM-I/R groups, the hearts were stabilized with KH buffer perfusion for 30 min, followed by global ischemia induction for 30 min by stopping the flow of KH buffer, and reperfusion for 60 min by restoring the KH buffer flow.

### 2.3. Induction of Diabetes Mellitus (DM)

Diabetes was induced in 6-week-old rats with a dose of 65 mg/kg b.wt. streptozotocin. After 72 h, the blood sample was collected from the orbital sinus of rats under anesthesia [10] and were checked for blood glucose using blood-glucose strips and AP Plus Glucometer. Rats with blood glucose >250 mg/dL were considered diabetic, and we maintained the diabetes condition for a further 18 weeks. Further, glycosylated hemoglobin was estimated in the venous blood (collected from tail vein) by the cation-exchange resin reaction method, using Monozymes’s Glycohemin kit (Monozyme India Ltd, Secunderabad, Telangana, India). The plasma was collected from blood via centrifuging the blood in EDTA-coated tubes at 3000 rpm for 5 min at 4 °C, and the supernatant was collected and stored as plasma. Plasma insulin was assessed by an enzyme-linked immunosorbent assay (ELISA) kit (Diagnostic Production Corporation, Oxfordshire, UK). This kit considers human insulin standard and labelled human insulin antibody, which cross-reacts with rat insulin. C-peptide analyses were performed using a radioimmunoassay (RIA) kit for rats supplied by Linco Research Laboratories, St. Charles, MO, USA.

### 2.4. I/R Injury Assessment

The changes in the cardiac performance were measured via the hemodynamic parameters. A balloon was inserted into the left ventricle of the mounted hearts, which was connected to a pressure transducer to measure the left ventricular pressure changes. The corresponding ventricular pressure changes were recorded continuously using Labchart Pro 8 software and Power Lab Data Acquisition System (AD Instruments, Sydney, Australia). The recorded left ventricular pressure was further used to derive the heart rate, left ventricular developed pressure (LVDP), and the maximum and minimum pressure derivative (dp/dt). Cardiac rate pressure product (RPP) was evaluated by multiplying the LVDP and cardiac heart rate to assess the cardiac recovery. The cardiac injury was assessed by measuring the infarct size of the hearts by staining the heart sections with 1.5% tetrazolium chloride (TTC) for 10 min, followed by formaldehyde fixation [11]. The area of infarct was measured by using Image J software (NIH, Bethesda, MD, USA) and reported as a percentage of infarct size. 

### 2.5. DNA Methylation Analysis

DNA was isolated from the cardiac tissue samples using a phenol/chloroform/isoamyl alcohol mixture (25:24:1) and precipitated with 100% ethanol as described elsewhere [12] Global DNA methylation was evaluated in the isolated cardiac DNA samples using MethylFlash™ Global DNA Methylation (5-mC) ELISA Easy Kit (Epigentek, Farmingdale, NY, USA), as per the kit instructions. Nuclear extract was separated from the cytosolic extract in rat heart samples, and DNMT activity was measured in the isolated nuclear extract using the EpiQuik™ DNMT (DNA methyltransferase) activity/inhibition assay ultra kit (#3010), as per the instructions in the kit. 

### 2.6. Gene Expression Analysis

Total RNA was isolated from the cardiac tissue using TRIzol reagent (Thermo Fisher Scientific, Waltham, MA, USA) and checked for purity. The isolated pure RNA was further converted into cDNA as per the kit protocol (Thermo Fisher Scientific, Waltham, MA, USA), and the mRNA expression of the genes was assessed by real-time PCR analysis (ABI 7500 (Applied Biosystems, Thermofisher, Waltham, CA, USA)) using DyNAmo Flash SYBR (Thermo Fisher Scientific). The mRNA expression of the genes involved in the process of DNA methylation (*DNMT1*, *DNMT 3A*, *DNMT3B*, *TET1*, *TET2*, *TET3*), mitochondrial biogenesis (*PGC1α*), mitochondrial transcription (*TFAM*), mitochondrial proliferation (*POLGA*), mitochondrial fission ((*MFN1*, *MFN2*), mitochondrial fusion (*MFF*, *FIS*, *DNM1*), and mitophagy (*PINK1*, *PARKIN*, *OPTN*) were assessed. Further the mRNA expression of the genes encoded by mitochondria involved in the mitochondrial complex I activity (*ND1*, *ND2*, *ND3*, *ND4*, *ND4L*, *ND5*, *ND6*), complex III activity (*Cyt B*), complex IV activity (*COX1*, *COX2*, *COX3*), and complex V activity (*ATP6*, *ATP8*)) were also assessed. The primer sequences of the genes are provided in Table 1. The mRNA expression of the genes were normalized with *GAPDH* (housekeeping gene) and the relative mRNA expression was calculated by using the Livak et al. method described elsewhere [13].

### 2.7. Mitochondrial Isolation and Mitochondrial Functional Evaluation

Mitochondria were isolated from the cardiac tissue samples as per the protocol mentioned in Palmer et al. [14], based on density–gradient separation centrifugation. In short, tissue was homogenized (10% *w*/*v*) in isolation buffer (Tris buffer (pH 7.5) containing 100 mM KCl, 40 mM Tris HCl, 40 mM MgCl_2_, 1 mM EDTA, 1 mM ATP) and then centrifuged at 600 g for 10 min at 4 °C. The resultant supernatant was further centrifuged at a higher speed of 6000 g for 10 min at 4 °C. The resultant mitochondrial pellet was then resuspended in the aforementioned isolation buffer containing 1.5% fatty-acid-free BSA and further centrifuged at 12,000 g for 10 min to yield the pure mitochondrial fraction as a pellet. The mitochondrial pellet was resuspended in storage buffer (10 mM Tris HCl (pH7.4) containing 200 mM sucrose, 70 mM mannitol, and 1 mM EDTA) and the concentration of the isolated mitochondrial protein was estimated using a Bradford assay with the Bradford reagent (BioRad) with bovine serum albumin as a standard. For the assessment of mitochondrial electron transport chain activities (ETC), the mitochondria were resuspended in hypotonic medium (25 mM K_2_PO_4_ pH-7.2, 5 mM MgCl_2_) and subjected to 3 cycles of freeze–thaw to disrupt the mitochondrial membranes. The ETC activities were the analyzed using a spectrophotometer with specific donor–acceptors, as described elsewhere [15]. ATP content was estimated in the mitochondrial samples using an ATP lite (Perkin Elmer, Waltham, MA, USA) kit, according to the manufacturer’s instructions. Briefly, lysis buffer was added to the homogenate/sample protein (100 µg) and shaken for 10 min. The substrate buffer (100 µL) was then added and incubated in the dark for 10 min. The luminescence intensity was captured using a Synergy H1 Multimode reader (BioTek, Winooski, VT, USA) as per the kit instructions.

### 2.8. Mitochondrial DNA Copy Number Estimation

The expression of the nuclear-encoded gene *β-actin* and mitochondrial-encoded gene *MT-ND1* was quantified from the extracted DNA samples, using SYBR green real-time PCR analysis. The ratio of the relative gene expression of the *MT-ND1* gene and the *β-actin* gene was calculated and presented as mitochondrial DNA copy number (mtDNA copy no.). The primer sequences of the genes are provided in Table 1.

### 2.9. Statistical Analysis

All data are represented as the mean ± SD. The data were analyzed using Graph Pad Prism 7.0 software (Graph Pad software, Inc., San Diego, CA, USA) for the significance between the groups with one-way ANOVA test, followed by post hoc analysis with Dunnett’s test. Correlation analysis was performed using the Pearson coefficient method.

## 3. Results

### 3.1. Basal-Level Changes in DM Rats

**Figure 1:**
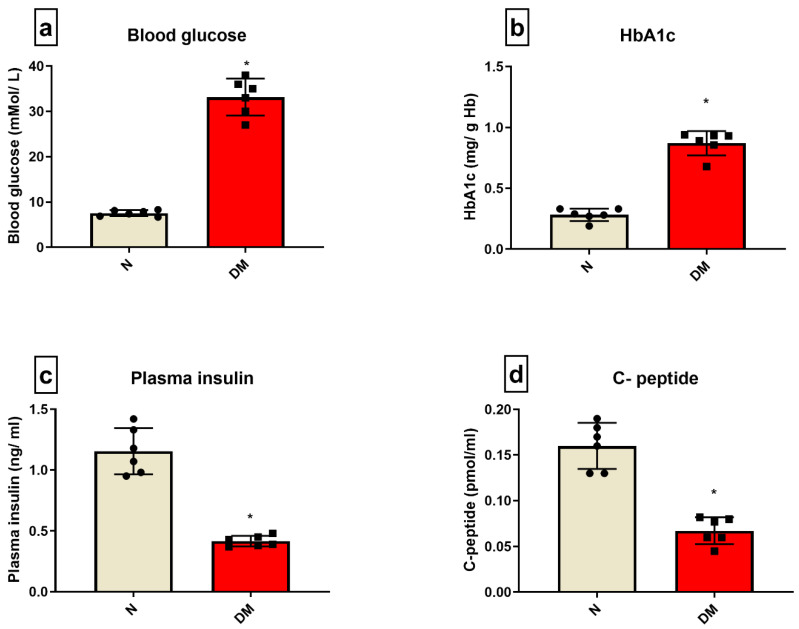
**DM characterization:** The characteristics of DM were evaluated in the rat blood by assessing the levels of (**a**) blood glucose; (**b**) HbA1C; (**c**) plasma insulin; (**d**) C-peptide. The graphs represent mean ± SD values. * *p* < 0.05 vs. *N*.

Figure 1 shows the basal-level changes in the blood chemistry of DM rats, wherein the DM animals showed an increase in blood glucose level and HbA1C level by 78% and 66%, respectively, and a decline in plasma insulin and C-peptide levels by 54% and 51%, respectively, from the sham control animal.

### 3.2. Hemodynamics of Diabetic Heart Deteriorated Significantly and Resulted in Higher Cardiac Injury

**Figure 2:**
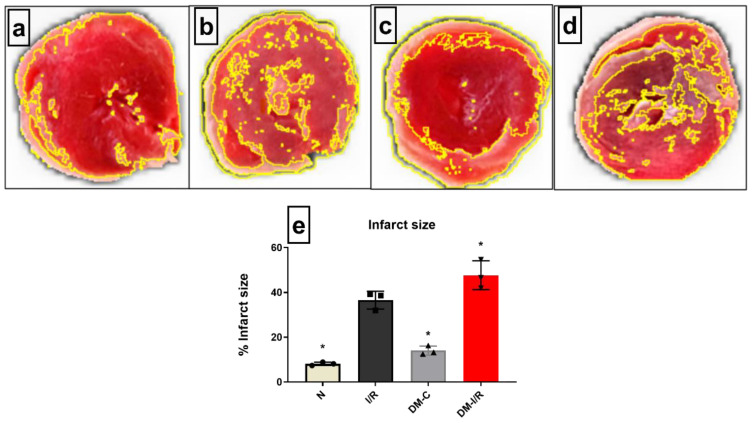
**Impact of DM on cardiac I/R injury:** The cardiac I/R injury was assessed in DM rat hearts by infarct size measurement of (**a**) normal; (**b**) I/R; (**c**) DM-C and (**d**) DM-I/R hearts. The presented images are representatives from the groups. (**e**) represents the infarct size percentage measurement. The graph represent mean ± SD values. * *p* < 0.05 vs. *N*.

Hemodynamic parameters like LVDP, RPP, and dp/dt were insignificantly deteriorated in the diabetic control hearts when compared with normal heart under normal perfusion (Table 2). The deteriorated hemodynamics of diabetic hearts surged during I/R, evident by a significant decrease in RPP and in the rate of relaxation and contraction by 43%, 42%, and 63%, respectively, from the DM-C hearts. In coherence with these results, the cardiac injury parameters like infarct size showed an increase by 65% (Figure 2) when compared with the DM-C rat hearts. On the other hand, the DM-C rat hearts did not exhibit a significant increase in infarct size from the normal heart.

### 3.3. DM Elevated DNA Hypermethylation Level and Augmented the Expression of Genes Involved in Methylation’s Impact on I/R

**Figure 3:**
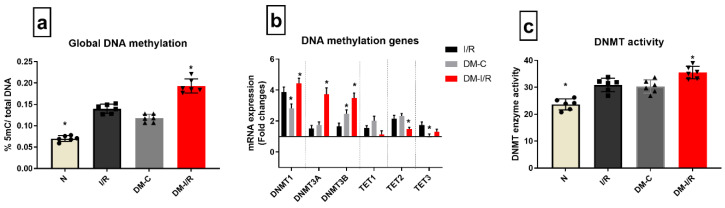
**Methylation changes in I/R challenged DM rat hearts:** The methylation changes in the DM I/R hearts were assessed from (**a**) global DNA methylation; (**b**) the mRNA expression of DNA methylation enzymes *DNMT1*, *3A*, *3B*, *TET1*, *2*, *3* (expressed as fold changes from normal); (**c**) DNMT enzyme activity. The graphs represent mean ± SD values. * *p* < 0.05 vs. *I*/*R*.

According to Figure 3a, the global DNA methylation level was elevated by 39% in DM-C hearts from sham, which was further increased by 42% in DM-I/R hearts when compared to DM-C. Diabetic sham rat hearts exhibited a significant increase in the expression of *DNMT1* and *3A* and *3B* gene expression by 2.8 and 2.4 folds, respectively, which was further elevated in DM-I/R hearts to 4.4, 3.7, and 3.4 folds, respectively (Figure 3b) compared to the sham hearts. In accordance with the upregulation of the DNMT gene, its activity in the myocardium was elevated in both DM-C and DM-I/R rat heart (Figure 3c). On the other hand, even though the TET 3 gene expression was upregulated in DM-C hearts, it did not show any significant variation in DM-I/R heart from the sham. The other TET genes did not show any significant variation in DM-C hearts.

In order to elucidate a relationship between DNA methylation and I/R injury, we conducted an association analysis and found a significant negative correlation between global DNA methylation level with both RPP (r = −0.7591, *p* = 0.043) and infarct size (r = −0.7048, *p* = −0.049) (Table 3).

### 3.4. DM Heart Deteriorated the Expression of ETC-Linked Genes and Mitochondrial Quality Control Genes That Negatively Imparted I/R-Associated Mitochondrial Dysfunction

**Figure 4:**
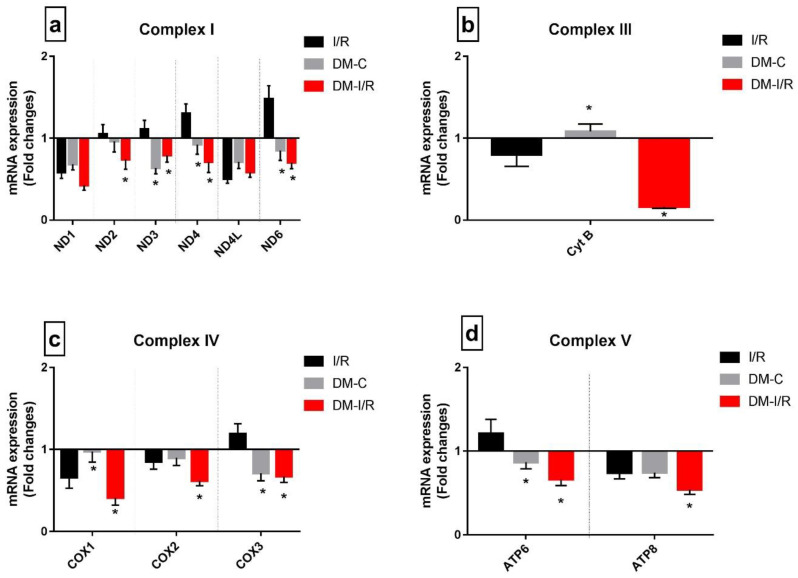
**Impact of DM on the expression of mitochondrial-encoded ETC genes during I/R:** The mRNA expression of mitochondrial-DNA-encoded genes are presented as fold changes from the normal group for (**a**) Complex I; (**b**) Complex III; (**c**) Complex IV; (**d**) Complex V. The graphs represent mean ± SD values. * *p* < 0.05 vs. *I*/*R*.

**Figure 5:**
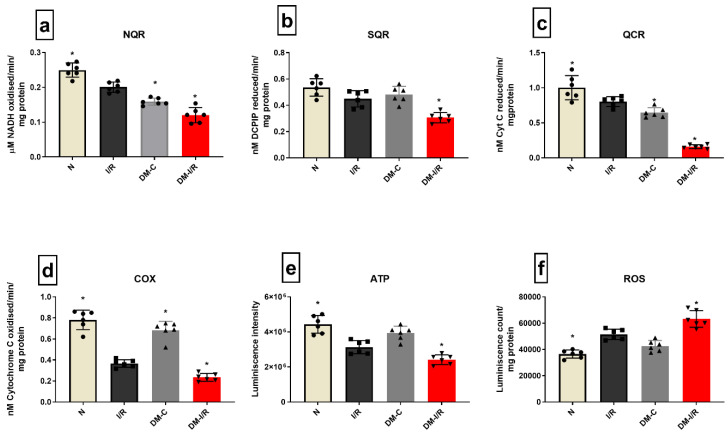
**Mitochondrial function assessment in DM hearts post I/R**—The effect of I/R on mitochondrial function in DM hearts was assessed by evaluating the enzyme activities of electron transport chain complexes (**a**) NQR (Complex I); (**b**) SQR (Complex II); (**c**) QCR (Complex III); (**d**) COX (Complex IV); and (**e**) ATP content; (**f**) ROS levels. The graphs represent mean ± SD values. * *p* < 0.05 vs. *I*/*R*.

Deteriorated mitochondrial bioenergetics function is one of the cardinal features of I/R pathology and is under the control of both nuclear and mitochondrial encoded genes. Figure 4 shows the gene expression of 13 mitochondrial-encoded ETC genes. Diabetic sham rat hearts showed a downregulation in all ETC genes except complex III encoding the *Cyt B* gene. I/R induction in diabetic rat hearts in DM-I/R group further downregulated all the ETC genes including *Cyt B* significantly when compared with the sham hearts except *ND1* (Figure 4). The downregulation of the ETC genes in diabetic I/R hearts in turn reduced the activities of ETC complexes like I, II, III, and IV, which declined by 38%, 37%, 75%, and 66%, respectively, along with declined ATP and increased ROS in diabetic I/R rat hearts when compared with DM-C hearts (Figure 5). However, a correlation study did not show any association between DNA methylation level and ETC genes

**Figure 6:**
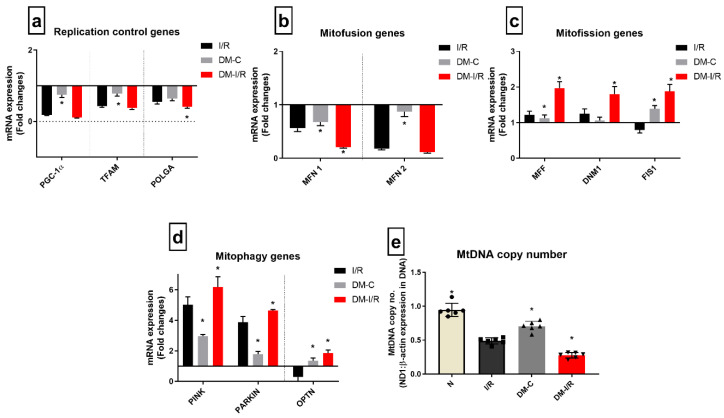
**Impact of diabetes on the expression of mitochondrial dynamics genes during I/R:** The mRNA expression of mitochondrial regulation control genes are presented as fold changes from the normal group for the processes of (**a**) mitochondrial replication; (**b**) mitofusion; (**c**) mitofission; (**d**) mitophagy; and the end effector (**e**) mitochondrial DNA copy number. The graphs represent mean ± SD values. * *p* < 0.05 vs. *I*/*R*.

Figure 6 shows the differential gene expression pattern of mitochondrial regulatory genes (mitochondrial biogenesis: *PGC1α*, mitochondrial replication: *TFAM* and *POLGA*, mitochondrial fission, fusion and mitophagy) genes in diabetic rat hearts.

DM-C rat heart exhibited decreased basal-level expression compared to normal heart in the mitochondrial replication control genes *PGC1α*, *TFAM*, and *POLGA*; mitofusion genes *MFN1* and *MFN2*; and the increased expression of the mitophagy genes *PINK*, *PARKIN*, and *OPTN* by 2.8-, 2-, 1.6-fold, respectively. Similarly, in DM I/R rat heart, the gene expression of mitophagy and mitofission genes were upregulated further significantly compared to DM-C (*PINK*, *PARKIN*, and *OPTN* genes by 6.1-, 5.2-, 2-, 3-fold, respectively, from I/R); while mitofusion genes and replication genes like *PGC1α* and *TFAM* were downregulated by 6.8- and 2.1-fold, respectively, compared to DM-C (Figure 6a).

In addition, the calculated mitochondrial copy number, which was significantly reduced in the DM sham compared to the normal, was further reduced in DM-I/R rat hearts. However, the I/R-associated mitochondrial copy number decline was similar in both normal and DM hearts subjected to I/R, but the absolute decrease was high in DM-C rat heart (Figure 6e).

Further, the correlation study results suggested that the expression levels of the three mRNA transcripts *PGC1α*, *TFAM*, and *POLGA* were negatively correlated with the DNA methylation (Pearson correlation coefficient r value = −0.9561, −0.8598, −0.9183, respectively) (Table 3).

## 4. Discussion

Cardiac recovery of the ischemic heart is generally assessed by measuring the hemodynamics and infarct size of the myocardium, which in turn depends on the functional activity of cardiac mitochondria, which regulates metabolic homeostasis. A battery of genes is involved in the regulation of metabolic homeostasis, that in turn is partly under the control of epigenetic regulation. Our previous study had reported that I/R can induce global DNA hypermethylation that resulted in the downregulation of the I/R-specific mitochondrial quality control genes and bioenergetic genes that promote mitochondrial dysfunction [16]. Recent reports demonstrated that the hypermethylation of global DNA and mitochondrial dysfunction co-exists in diabetic rat heart [9]. However, the consequential effect of these changes at the gene level during cardiac I/R injury is yet to be explored and is addressed in the current study.

In the present study, diabetic rat hearts exhibited significant elevation in global DNA methylation with a subsequent increase in DNMT gene expression in the basal tissue level. The I/R induction of these rats further surged the global DNA hypermethylation level, along with a higher infarct size and severely compromised cardiac hemodynamics. Interestingly, I/R-associated cardiac injury and hemodynamic changes were similar in heart with and without diabetes. However, the higher magnitude of injury and deteriorated hemodynamics in DM I/R hearts suggest that basal-tissue changes contribute negatively to the pathology. In order to confirm this, we utilized both the isolated rat heart model (absence of neuroharmonal influence) and the LAD model (presence of neuroharmonal influence), wherein we found a similar magnitude of injury in DM-I/R rat hearts, emphasizing the significance of basal-level changes in tissue that play a critical role in determining the overall cardiac I/R associated damage.

Since our main objective was to explore DM-associated modifications at the gene level in the myocardium, we extensively analyzed the isolated rat heart subjected to I/R. The higher infarct size in the DM-I/R rat heart may be due to the additive effect in basal diabetic rat heart with I/R induction. Further, we analyzed the correlation between RPP and infarct size with the global DNA methylation level and found a negative correlation with both the parameters, suggesting that global DNA hypermethylation changes indeed influence cardiac physiology and the cardiac injury events. In our previous publication, we demonstrated that reducing DNA hypermethylation can improve cardiac physiology and limit the infarct size.

More than 2000 mitochondrial proteins are involved in the overall cardiac function. Thus, the dysfunction of these proteins by I/R-associated free radicals or inhibitors is critical in determining the I/R pathology. In fact, the gene regulation of these proteins by epigenetic control was recently exhibited as a therapeutic target in the management of I/R. In the present study, we measured the major mitochondrial gene expression involved in I/R with respect to the basal DM tissue and DM heart subjected to I/R. Further, we performed a correlation study of these genes with RPP and infarct size. It is well known from the literature that a single gene may be under the control of different epigenetic tags, and thus, we believed global DNA change will be more reliable data compared to gene-specific methylation, due to the involvement of multiple mitochondrial genes in the pathological event of I/R. These multiple mitochondrial genes are linked to different processes in the mitochondria that make it heathy. Accordingly, we evaluated the expression of genes involved in bioenergetics, mitophagy, mitofusion and fission, mitochondria replication, translation, and biogenesis and performed a correlation analysis with global DNA methylation.

Earlier studies have shown that one of the consequential effects of elevated *DNMT1* and high DNA methylation may be the deterioration of mitochondrial function, as few studies indicate that DNA methylation can control the expression of nuclear-encoded mitochondrial genes like *PGC1α* [17], *TFAM* [18], and *POLGA* [19]. These genes are critical for the mitochondrial bio-energetic functions, transcription, translation, and replication of cellular mitochondria.

In diabetic rat heart, we noted deteriorated mitochondrial bioenergetics function, and a previous study indicate the dysregulation of *PGC1α* and miRNA as one of the reasons [20]. Mitochondrial bioenergetics functions were under the control of nuclear-encoded genes like *PGC1α*, the master regulator of mitochondrial respiration and biogenesis and genes encodes for different subunits of the respirasome [21]. In addition, 13 mitochondrial-encoded genes regulate the assembly and function of the mitochondrial super complex structure for respiration [22]. Our results in Figure 6 suggest significant downregulation of the *PGC1α* gene and all 13 of the ETC genes except *COX2* in diabetic rat heart (Figure 4). Subsequent analysis of ETC enzyme activity showed significantly reduced activities of Complex I, III, and IV, indicating compromised mitochondrial function in diabetic heart. Further, we checked the mitochondrial DNA copy number to explain the defect in mitochondrial function in diabetic heart. Based on the results given in Figure 6e, we found a significant decline in the mitochondrial DNA copy number in diabetic rat heart, which was in agreement with the previous studies [23]. This reduced mitochondrial function may be a consequential effect from the mutation of mitochondrial DNA that occurs during diabetes mellitus [24]. However, the possibility of reduced mitochondrial DNA replication that may be responsible for the low mitochondrial DNA copy number cannot be ruled out in diabetes mellitus. In the present study, we found a significant downregulation of *POLGA* gene expression that is responsible for the replication. Reports suggest that low mitochondrial copy number is also associated with the mitophagy that may also contribute to its decline, especially during starvation [25], which is a deprived cellular energetic state. Accordingly, we found significant upregulation of the mitophagy genes like *PINK*, *PARKIN*, and *OPTN* (Figure 6d). In addition, *OPTN*, the primary autophagy receptor showed a three-fold increase in its expression in diabetic heart. Early studies have reported the link between *OPTN* expression and the progression of diabetes [26], wherein its expression can be induced by TNF-α and interferons. Among the different mitochondrial dynamics genes like *MFN-1*, *MFN2*, *FIS 1*, *DNM1*, and *MFF*, the fusion genes like *MFN-1* and *MFN-2* were downregulated, while fission genes like *FIS1*, *DNM 1*, and *MFF* were upregulated (Figure 6c). Many studies in the literature confirmed that the hyperglycemia and hyperlipidemia that develop during diabetes can induce mitochondrial fission in heart and thereby facilitate the ROS level in mitochondria [27].

Based on the results obtained in the present study, during diabetes mellitus, the quality of cardiac mitochondria becomes deteriorated and may facilitate the progression of the pathology associated with diabetes via the production of ROS, suppressing ETC activity, ATP level, and mitochondrial DNA copy number. DNA methylation is reported to be associated with the regulation of most of these genes that determine the quality [28]. Hence the elevated DNMT 1 and DNA methylation level was further statistically analyzed for a possible association. Based on the data, we found a strong negative correlation of DNA methylation with *PGC1α*, *TFAM*, and *POLGA*. A recent study has reported the presence of DNA methylation in the promoter region of *PGC1α* [29] and *POLGA* [30]. This suggests the possible interaction of *DNMT1* with promoter of PGC1α in diabetic rat hearts, resulting in the reduced mRNA expression of *PGC1α*, subsequent mitochondrial DNA mutation, and dysregulated redox balance in the mitochondria, which was reflected in the low bioenergetics function and elevated release of ROS.

Stress can modify the DNA methylation, and that may change gene expression and contribute to disease phenotypes [31]. In order to understand the I/R-associated stress-mediated alterations in DNA methylation in diabetic heart (expressed higher oxidative stress) and its effect on mitochondrial function, we analyzed the level of DNA methylation and the gene expression of *DNMT1*. Unlike diabetic heart, the diabetic I/R heart showed a severe decline in mitochondrial function. This may be due to the striking low level of the gene expression of the nuclear-encoded mitochondrial functional regulatory genes *PGC1α*, *TFAM*, and *POLGA* at the basal level itself. Furthermore, the mitochondrial encoded genes that regulate the synthesis of ETC subunits were downregulated significantly in the DM-I/R heart, accounting for the reduced bioenergetics function in diabetic I/R hearts. Furthermore, I/R downregulated MFN-1 and MFN-2 and upregulated *PINK*, *PARKIN*, and *OPTN*, significantly from the diabetic control rat heart. These alterations in the mitochondrial genes emphasize the further deterioration of mitochondrial quality in cardiac I/R heart during diabetes. Next, we did a correlation analysis between mitochondrial genes and global DNA methylation. Similar to diabetic rat heart, upon I/R exposure, *POLGA*, *TFAM*, *PGC1α*, and additionally, *MT-ND1* showed a negative correlation with global DNA methylation.

## 5. Conclusions

Based on the results obtained in the present study, we found that diabetic rat heart induced the hypermethylation of global DNA and mitochondrial dysfunction by upregulating mitophagy and mitofission genes and downregulating mitochondrial replication, ETC and mitochondrial fusion genes even at the basal level. In addition, diabetic rat heart exhibited a similar pattern of changes even in I/R conditions but with a higher degree of methylation and mitochondrial dysfunction, resulting in increased I/R injury. Importantly, the I/R associated changes were similar in both non-diabetic and diabetic I/R hearts. However, the pre-existing changes in DNA methylation and mitochondrial dysfunction in the basal tissue under DM conditions contributed to its enhanced injury during I/R.

## Figures and Tables

**Table 1:** **Primer sequence details:** The forward and reverse primer sequences of the genes used for real-time PCR analysis are presented.

S. No	Gene	Gene Accession No.	Forward Primer	Reverse Primer
1	*GAPDH*	NM_017008.4	5′-GCGAGATCCCGCTAACATCA-3′	5′-CTCGTGGTTCACACCCATCA-3′
2	*PGC 1α*	NM_031347.1	5′-GAGGGACGAATACCGCAGAG-3′	5′-CTCTCAGTTCTGTCCGCGTT-3′
3	*Dnm1*	NM_080689.5	5′-TTGCCCTCTTCAACACTGAGC-3′	5′-ATGAAGCTGTCAGAGCCGTT-3′
4	*Parkin*	NM_020093.1	5′-AGTTTGTCCACGACGCTCAA-3′	5′-CAGAAAACGAACCCACAGCC-3′
5	*MFN1*	NM_138976.1	5′-TGACTTGGACTACTCGTGCG-3′	5′-GGCACAGTCGAGCAAAAGTG-3′
6	*MFN2*	NM_130894.4	5′-CTCTGTGCTGGTTGACGAGT-3′	5′-TCGAGGGACCAGCATGTCTA-3′
7	*DRP1*	AF019043.2	5′-TGGAAAGAGCTCAGTGCTGG-3′	5′-TCAACTCCATTTTCTTCTCCTGT-3′
8	*MFF*	NM_001271284.1	5′-GAAAACACCTCCACGTGTGC-3′	5′-CTGCTCGGATCTCTTCGCTT-3′
9	*FIS*	NM_001105919.2	5′-CCAGAGATGAAGCTGCAAGGA-3′	5′-TTCCTTGAGCCGGTAGTTGC-3′
10	*PINK1*	NM_001106694.1	5′-TGTATGAAGCCACCATGCCC-3′	5′-TCTGCTCCCTTTGAGACGAC-3′
11	*TFAM*	NM_031326.2	5′-GTTGCTGTCGCTTGTGAGTG-3′	5′-GTCTTTGAGTCCCCCATCCC-3′
12	*β-actin*	NM_031144.3	5′-GTGTGGTCAGCCCTGTAGTT-3′	5′-CCTAGAAGCATTTGCGGTGC-3′
13	*POLG1*	NM_053528.1	5′-CTTTGGGCTCCAGCTTGACT-3′	5′-TGGAGAAAATGCTTGGCACG-3′
14	*ND1*	NC_001665.2	5′-CCACCGCGGTCATACGATTA-3′	5′-AGGGCTAAGCATAGTGGGGT-3′
15	*CYTB*	NC_001665	5′-ACAAAATCCCATTCCATCCA-3′	5′-GTTGGGAATGGAGCGTAGAA-3′
16	*ND6*	NC_001665.2	5′-ATCCGGAAACTTGAGGGTCT-3′	5′-CCCAGCCACCACTATCATTC-3′
17	*ND5*	NC_001665.2	5′-ATTGCAGCCACAGGAAAATC-3′	5′-TGGTGATTGCACCAAGACAT-3′
18	*ND4L*	NC_001665.2	5′-GTACTTTTATATTTCGCTCCCACT-3′	5′-CGCAGGCTGCAAAAACTAGA-3′
19	*ND3*	NC_001665.2	5′-TGCATTCTGATTGCCTCAAA-3′	5′-TGGGAGGGGGAGTAGTAAGG-3′
20	*COX3*	NC_001665.2	5′-AGCCCATGACCACTAACAGG-3′	5′-TGGCCTTGGTATGTTCCTTC-3′
21	*ATP6*	NC_001665.2	5′-ACACCAAAAGGACGAACCTG-3′	5′-AGAATTACGGCTCCTGCTCA-3′
22	*ATP8*	NC_001665.2	5′-ACACCAAAAGGACGAACCTG-3′	5′-AGAATTACGGCTCCTGCTCA-3′
23	*COX2*	NC_001665.2	5′-GCTTACAAGACGCCACATCA-3′	5′-GAATTCGTAGGGAGGGAAGG-3′
24	*COX1*	NC_001665.2	5′-AATTGGAGGCTTCGGAAACT-3′	5′-CTGTTCCAGCTCCAGCTTCT-3′
25	*ND2*	NC_001665.2	5′-AAAAAGCCCACGATCAACTG-3′	5′-GGGAATTCCTTGGGTGACTT-3′
26	*ND4*	NC_001665.2	5′-CCCACTCTTAATTGCCCTCA-3′	5′-CGTGGGCTTTTGGTAATCAT-3′
27	*DNMT1*	NM_053354.3	5′CGGATTGTCGGATAAAAGA3′	5′GCTTCCTCATCGCTCCAGTA3′
28	*DNMT 3A*	NM_001003958.1	5′-GGAGAGGAAAGGGAGAGAGG-3′	5′-AGGGATGGTGCTGGTGAGAC-3′
29	*DNMT3B*	NM_001396349.1	5′-AAACCCAACAACAAGCAACC-3′	5′-ACATCAGAAGCCATCCGTTC-3′
30	*TET1*	XM_039099324.1	5′-TATATGGCTGTGCTGTGCTGCCCAA-3′	5′-CGATGGGCCATTGCTTGATG-3′
31	*TET2*	XM_006233347.4	5′-TGTTGTCAGGGTGAGAATCCAG-3′	5′-CCTGTAGGCATCAGGTGCAA-3′
32	*TET3*	XM_008763094.3	5′-CCCTTGCCTGAAGCATCTCA-3′	5′-GCCGAGGTACCATTCCCAAA-3′

**Table 2:** **Effect of I/R on cardiac function in diabetic hearts:** The impact of diabetes on cardiac I/R were evaluated by the hemodynamic changes LVDP (left ventricular diastolic pressure); RPP (rate pressure product = heart rate × LVDP) and ±dp/dt maximum/minimum force of contraction. The data are represented as mean ± SEM. * *p* < 0.05 vs. *I*/*R*.

Groups	LVDP (×10 mmHg)	RPP (mmHg × Beats/Min × 10^4^)	(−dp/dt) (×10 mmHg)	(+dp/dt) (×10 mmHg)
N	9.3 ± 1.6 *	2.5 ± 0.21 *	71.5 ± 9.3 *	98.7 ± 11.8 *
I/R	5.2 ± 0.8	1.4 ± 0.01	43.4 ± 4.2	64.4 ± 5.6
DM-C	7.2 ± 1.5 *	1.9 ± 0.21 *	58.7 ± 7.9 *	89.3 ± 10.2 *
DM-I/R	3.5 ± 1.3 *	0.8 ± 0.11 *	33.9 ± 4.9 *	31.8 ± 4.2 *

**Table 3:** Correlation data of global DNA methylation with the injury and mitochondrial function during cardiac I/R in diabetic rats: Pearson correlation analysis of global DNA methylation with the cardiac injury and hemodynamic parameters and the mitochondrial genes.

Parameter	Correlation Coefficient r-Value	*p*-Value
RPP	**−0.7591**	**0.043**
Infarct size	**−0.7048**	**0.049**
**Gene Expression**		
*PGC 1α*	**−0.9561**	**0.044**
*Dnm1*	−0.4591	0.234
*Parkin*	−0.3890	0.389
*MFN1*	−0.3124	0.445
*MFN2*	−0.5674	0.223
*DRP1*	−0.5542	0.356
*MFF*	−0.3998	0.149
*FIS*	−0.6565	0.111
*PINK1*	−0.7054	0.456
*TFAM*	**−0.8598**	**0.038**
*POLGA*	**−0.9183**	**0.045**
*ND1*	−0.2804	0.494
*CYTB*	−0.4409	0.567
*ND6*	−0.5367	0.445
*ND5*	−0.6498	0.167
*ND4L*	−0.7988	0.184
*ND3*	−0.9045	0.234
*COX3*	−0.5687	0.324
*ATP6*	−0.6789	0.183
*ATP8*	−0.6482	0.147
*COX2*	−0.5408	0.257
*COX1*	−0.6567	0.378
*ND2*	−0.7874	0.339
*ND4*	−0.8675	0.211

## Data Availability

Data will be available upon reasonable request.

## References

[1] Raciti G.A., Desiderio A., Longo M., Leone A., Zatterale F., Prevenzano I., Miele C., Napoli R., Beguinot F. (2021). DNA Methylation and Type 2 Diabetes: Novel Biomarkers for Risk Assessment?. Int. J. Mol. Sci..

[2] Fernández-Sanlés A., Sayols-Baixeras S., Subirana I., Sentí M., Pérez-Fernández S., de Castro Moura M., Esteller M., Marrugat J., Elosua R. (2021). DNA methylation biomarkers of myocardial infarction and cardiovascular disease. Clin. Epigenet..

[3] Marumo T., Yagi S., Kawarazaki W., Nishimoto M., Ayuzawa N., Watanabe A., Ueda K., Hirahashi J., Hishikawa K., Sakurai H. (2015). Diabetes Induces Aberrant DNA Methylation in the Proximal Tubules of the Kidney. J. Am. Soc. Nephrol..

[4] Chen Y.T., Liao J.W., Tsai Y.C., Tsai F.J. (2016). Inhibition of DNA methyltransferase 1 increases nuclear receptor subfamily 4 group A member 1 expression and decreases blood glucose in type 2 diabetes. Oncotarget.

[5] Hausenloy D.J., Yellon D.M. (2013). Myocardial ischemia-reperfusion injury: A neglected therapeutic target. J. Clin. Investig..

[6] Endres M., Meisel A., Biniszkiewicz D., Namura S., Prass K., Ruscher K., Lipski A., Jaenisch R., Moskowitz M.A., Dirnagl U. (2000). DNA methyltransferase contributes to delayed ischemic brain injury. J. Neurosci..

[7] Zhao Y., Ding C., Xue W., Ding X., Zheng J., Gao Y., Xia X., Li S., Liu J., Han F. (2017). Genome-wide DNA methylation analysis in renal ischemia reperfusion injury. Gene.

[8] Boovarahan S.R., Kurian G.A. (2021). Preconditioning the rat heart with 5-azacytidine attenuates myocardial ischemia/reperfusion injury via PI3K/GSK3β and mitochondrial K(ATP) signaling axis. J. Biochem. Mol. Toxicol..

[9] Bansal A., Pinney S.E. (2017). DNA methylation and its role in the pathogenesis of diabetes. Pediatr. Diabetes.

[10] Parasuraman S., Raveendran R., Kesavan R. (2010). Blood sample collection in small laboratory animals. J. Pharmacol. Pharmacother..

[11] Ferrera R., Benhabbouche S., Bopassa J.C., Li B., Ovize M. (2009). One hour reperfusion is enough to assess function and infarct size with TTC staining in Langendorff rat model. Cardiovasc. Drugs Ther..

[12] Pearson H., Stirling D., Bartlett J.M.S., Stirling D. (2003). DNA Extraction from Tissue. PCR Protocols.

[13] Livak K.J., Schmittgen T.D. (2001). Analysis of relative gene expression data using real-time quantitative PCR and the 2(-Delta Delta C(T)) Method. Methods.

[14] Palmer J.W., Tandler B., Hoppel C.L. (1977). Biochemical properties of subsarcolemmal and interfibrillar mitochondria isolated from rat cardiac muscle. J. Biol. Chem..

[15] Barrientos A., Fontanesi F., Díaz F. (2009). Evaluation of the mitochondrial respiratory chain and oxidative phosphorylation system using polarography and spectrophotometric enzyme assays. Curr. Protoc. Hum. Genet..

[16] Boovarahan S.R., Kurian G.A. (2022). Investigating the role of DNMT1 gene expression on myocardial ischemia reperfusion injury in rat and associated changes in mitochondria. Biochim. Biophys. Acta Bioenerg..

[17] Krämer A.I., Handschin C. (2019). How Epigenetic Modifications Drive the Expression and Mediate the Action of PGC-1α in the Regulation of Metabolism. Int. J. Mol. Sci..

[18] Gemma C., Sookoian S., Dieuzeide G., García S.I., Gianotti T.F., González C.D., Pirola C.J. (2010). Methylation of TFAM gene promoter in peripheral white blood cells is associated with insulin resistance in adolescents. Mol. Genet. Metab..

[19] Stoccoro A., Coppedè F. (2021). Mitochondrial DNA Methylation and Human Diseases. Int. J. Mol. Sci..

[20] Caravia X.M., Fanjul V., Oliver E., Roiz-Valle D., Morán-Álvarez A., Desdín-Micó G., Mittelbrunn M., Cabo R., Vega J.A., Rodríguez F. (2018). The microRNA-29/PGC1α regulatory axis is critical for metabolic control of cardiac function. PLoS Biol..

[21] Liang H., Ward W.F. (2006). PGC-1alpha: A key regulator of energy metabolism. Adv. Physiol. Educ..

[22] Kotrys A.V., Szczesny R.J. (2019). Mitochondrial Gene Expression and Beyond-Novel Aspects of Cellular Physiology. Cells.

[23] Memon A.A., Sundquist J., Hedelius A., Palmér K., Wang X., Sundquist K. (2021). Association of mitochondrial DNA copy number with prevalent and incident type 2 diabetes in women: A population-based follow-up study. Sci. Rep..

[24] Maassen J.A., ‘t Hart L.M., van Essen E., Heine R.J., Nijpels G., Jahangir Tafrechi R.S., Raap A.K., Janssen G.M., Lemkes H.H. (2004). Mitochondrial diabetes: Molecular mechanisms and clinical presentation. Diabetes.

[25] Medeiros T.C., Graef M. (2019). Autophagy determines mtDNA copy number dynamics during starvation. Autophagy.

[26] Chen K., Dai H., Yuan J., Chen J., Lin L., Zhang W., Wang L., Zhang J., Li K., He Y. (2018). Optineurin-mediated mitophagy protects renal tubular epithelial cells against accelerated senescence in diabetic nephropathy. Cell Death Dis..

[27] Kaludercic N., Di Lisa F. (2020). Mitochondrial ROS Formation in the Pathogenesis of Diabetic Cardiomyopathy. Front. Cardiovasc. Med..

[28] D’Aquila P., De Rango F., Guarasci F., Mandalà M., Corsonello A., Bellizzi D., Passarino G. (2020). Multi-Tissue DNA Methylation Remodeling at Mitochondrial Quality Control Genes According to Diet in Rat Aging Models. Nutrients.

[29] Santos J.L., Krause B.J., Cataldo L.R., Vega J., Salas-Pérez F., Mennickent P., Gallegos R., Milagro F.I., Prieto-Hontoria P., Riezu-Boj J.I. (2020). PPARGC1A Gene Promoter Methylation as a Biomarker of Insulin Secretion and Sensitivity in Response to Glucose Challenges. Nutrients.

[30] Tewari S., Zhong Q., Santos J.M., Kowluru R.A. (2012). Mitochondria DNA replication and DNA methylation in the metabolic memory associated with continued progression of diabetic retinopathy. Investig. Ophthalmol. Vis. Sci..

[31] Muka T., Koromani F., Portilla E., O’Connor A., Bramer W.M., Troup J., Chowdhury R., Dehghan A., Franco O.H. (2016). The role of epigenetic modifications in cardiovascular disease: A systematic review. Int. J. Cardiol..

